# A Critical Role for Neurofascin in Regulating Action Potential Initiation through Maintenance of the Axon Initial Segment

**DOI:** 10.1016/j.neuron.2011.02.021

**Published:** 2011-03-10

**Authors:** Barbara Zonta, Anne Desmazieres, Arianna Rinaldi, Steven Tait, Diane L. Sherman, Matthew F. Nolan, Peter J. Brophy

**Affiliations:** 1 Centre for Neuroregeneration, University of Edinburgh, Edinburgh EH16 4SB, UK; 2 Centre for Integrative Physiology, University of Edinburgh, Edinburgh EH8 9XD, UK

## Abstract

The axon initial segment (AIS) is critical for the initiation and propagation of action potentials. Assembly of the AIS requires interactions between scaffolding molecules and voltage-gated sodium channels, but the molecular mechanisms that stabilize the AIS are poorly understood. The neuronal isoform of Neurofascin, Nfasc186, clusters voltage-gated sodium channels at nodes of Ranvier in myelinated nerves: here, we investigate its role in AIS assembly and stabilization. Inactivation of the *Nfasc* gene in cerebellar Purkinje cells of adult mice causes rapid loss of Nfasc186 from the AIS but not from nodes of Ranvier. This causes AIS disintegration, impairment of motor learning and the abolition of the spontaneous tonic discharge typical of Purkinje cells. Nevertheless, action potentials with a modified waveform can still be evoked and basic motor abilities remain intact. We propose that Nfasc186 optimizes communication between mature neurons by anchoring the key elements of the adult AIS complex.

## Highlights

► The AIS and the node of Ranvier are assembled by distinct mechanisms ► The neuronal isoform of Neurofascin, Nfasc186, prevents breakdown of the AIS ► Selective disruption of the AIS abolishes spontaneous discharge in Purkinje cells ► The AIS is not essential for evoked action potentials but modulates their waveform

## Introduction

Initiation of action potentials is fundamental to signaling in vertebrate nervous systems. In mammalian neurons, the site of initiation of the action potential is believed to be the axon initial segment (AIS), a specialized region located between the axon hillock and the myelin sheath in myelinated axons (Palay et al., 1968). Hence, the properties of the AIS are likely to determine how a neuron responds to excitatory and inhibitory synaptic inputs. Recent experiments suggest that the composition and topographical organization of the initial segment are dynamically and precisely organized (Colbert and Pan, 2002; Fleidervish et al., 2010; Grubb and Burrone, 2010a; Hu et al., 2009; Kole et al., 2008; Kuba et al., 2010). The relatively low threshold for initiation of action potentials at the AIS is believed to rely on the high density and specialized gating properties of voltage-gated sodium channels (Na_v_). Clustering of these sodium channels during development depends on the correct targeting of AnkyrinG to the AIS (Hedstrom et al., 2008; Zhou et al., 1998). However, the molecular mechanisms that maintain an appropriate configuration of the AIS in adult neurons in vivo are poorly understood.

In addition to Na_v_ channels and AnkyrinG, the AIS contains a high density of proteins also found in nodes of Ranvier. These include voltage-gated potassium channels (K_v_) (Clark et al., 2009), the scaffolding protein βIV-Spectrin and the cell-adhesion molecules Neurofascin186 (Nfasc186) and neuron-glia related cell-adhesion molecule (NrCAM) (Rasband, 2010). In contrast to nodes of Ranvier, assembly of these molecules at the axon initial segment does not require glial derived cues (Dzhashiashvili et al., 2007; Rasband, 2010). Moreover, although once considered a stable neuronal compartment, recent studies have shown that the AIS can change its position in an activity-dependent manner (Grubb and Burrone, 2010a, 2010b; Kuba et al., 2010). It has also become increasingly clear that the molecular composition of the AIS varies between different cell-types (Lorincz and Nusser, 2008) and within its distal and proximal compartments (Hu et al., 2009; Van Wart et al., 2007). This heterogeneity may contribute to the specificity with which neurons initiate and shape action potentials (Nusser, 2009).

Despite its probable importance, our understanding of the molecular mechanisms of assembly, maintenance and plasticity of the AIS is still limited. AnkyrinG has been proposed as the master organizer of the AIS (Dzhashiashvili et al., 2007; Sobotzik et al., 2009). During development this scaffold protein appears to be targeted to the domain earlier than other proteins (Jenkins and Bennett, 2001), where it is believed to bind conserved motifs in Na_v_ channels (Garrido et al., 2003; Lemaillet et al., 2003; Pan et al., 2006), sodium channel β1 subunits in a phosphotyrosine-dependent manner (Malhotra et al., 2002), K_v_ channels (Pan et al., 2006; Rasmussen et al., 2007), Neurofascin and NrCAM (Boiko et al., 2007; Davis and Bennett, 1994; Garver et al., 1997; Zhang and Bennett, 1998), and βIV-Spectrin (Yang et al., 2007). Further support for this view comes from the failure of the Purkinje cell AIS to assemble in mice that lack cerebellar AnkyrinG during development (Jenkins and Bennett, 2001; Zhou et al., 1998). Knockdown studies also show that AnkyrinG is required for assembly and maintenance of the AIS molecular complex in cultured hippocampal neurons (Hedstrom et al., 2007, 2008). Deletion of the Ankyrin-interactor βIV-Spectrin leads to redistribution of AIS proteins but does not abolish the AIS (Lacas-Gervais et al., 2004; Yang et al., 2004). Knocking down Na_v_ channels also disrupts the AIS molecular complex in cultured spinal motor neurons (Xu and Shrager, 2005), but not in other types of neuron (Hedstrom et al., 2007).

It is not known if distinct molecular mechanisms are required for stable maintenance of the AIS in vivo following maturation of the nervous system by comparison with those involved in assembly of the AIS during development. Indeed, the role of both Neurofascin and NrCAM at the AIS is still unclear. In contrast to its pioneer role in node of Ranvier formation in the PNS and CNS (Dzhashiashvili et al., 2007; Eshed et al., 2005; Feinberg et al., 2010; Koticha et al., 2006; Sherman et al., 2005; Zonta et al., 2008), Nfasc186 appears to be dependent upon AnkyrinG binding for its localization to the AIS through a FIGQY motif in its cytoplasmic domain (Davis and Bennett, 1994; Dzhashiashvili et al., 2007; Lemaillet et al., 2003). Further, RNAi knockdown of NrCAM and Nfasc186 has suggested that they are not required for the assembly of the AIS in cultured hippocampal neurons, but rather that Nfasc186 has a role in targeting the extracellular matrix (ECM) protein Brevican (Hedstrom et al., 2007). GABAergic innervation by basket cell axons to the Purkinje cell AIS, known as pinceau synapses, also appears to be directed by Nfasc186, through a mechanism that in turn depends on AnkyrinG (Ango et al., 2004).

We have used an in vivo approach to ask if Nfasc186 has an active role in AIS structure and function. Our study shows that Nfasc186 is not required for the assembly of the AIS during development, although it is required to target NrCAM. In contrast, using an inducible conditional strategy to ablate Neurofascin biosynthesis in adult neurons, we show that loss of Nfasc186 causes breakdown of the AIS complex and impairment of normal action potential initiation in Purkinje cells. Surprisingly, Nfasc186 is much more stable in the nodal complex, and nodes of Ranvier are much less susceptible to disintegration. This has allowed us to study the functional consequences of AIS disruption in the presence of intact nodes of Ranvier in vivo. Our results show that AIS disruption due to loss of Nfasc186 prevents spontaneous action potential firing by cerebellar Purkinje cells, modifies the waveform of evoked action potentials, and leads to an impairment in motor performance. Our data suggest that the molecular mechanisms for stabilization of the AIS in adult neurons in vivo are distinct from the mechanisms used for assembly of the AIS in developing neurons. We propose a dynamic model for maintenance of the mature AIS, whereby Nfasc186 is constitutively required for anchoring of new protein components to the AIS complex.

## Results

### AIS Assembly without Neurofascin In Vivo

**Figure 1:**
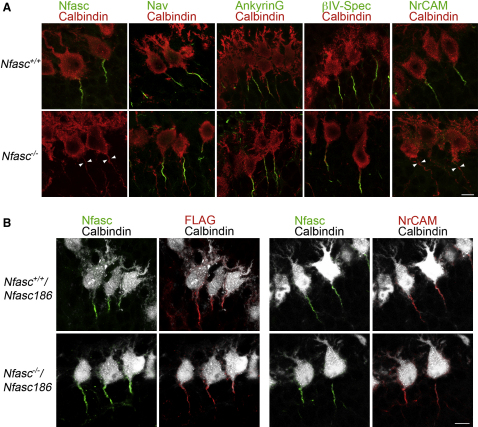
Nfasc Is Not Required for AIS Assembly (A) Immunofluorescence analysis of Calbindin-positive Purkinje cells in sagittal cryostat sections of cerebella from P6 wild-type littermates and *Nfasc^−/−^* mice shows no difference in the localization of voltage-gated sodium (Na_v_) channels, AnkyrinG, and βIV-Spectrin at the AIS. However, NrCAM fails to localize at the AIS in the mutant (arrowheads point to AIS location). Scale bar, 10 μm. (B) Immunofluorescence of Calbindin-positive Purkinje cells in sagittal cryostat sections from P6 *Nfasc^+/+^/Nfasc186* and *Nfasc^−/−^/Nfasc186* mice shows that Nfasc186Flag is correctly targeted to the AIS and that its expression on a *Nfasc* null background can restore NrCAM to the AIS. Scale bar, 10 μm. See also Figure S1.

To test whether proteins known to be constituents of the initial segment complex could cluster appropriately in the absence of the Neurofascins (the neuronal isoform Nfasc186 and the glial isoform Nfasc155), we examined the cerebella of wild-type and Neurofascin null mice at P6. NrCAM was the only component of the AIS complex found to be affected in mutant Purkinje cells (Figure 1A), and the number of Purkinje cells positive for NrCAM was reduced from 93.0% ± 1.3% to 7.4% ± 0.1% (mean values ± SEM, n = 3, 40 cells per animal, p < 0.0001, unpaired Student's t test). In order to establish if the presence of NrCAM at the AIS was dependent on the neuronal isoform of Neurofascin, Nfasc186, we generated transgenic mice expressing FLAG-tagged Nfasc186 on a Neurofascin null background. The transgenic Nfasc186 was targeted appropriately and rescued NrCAM at the AIS (Figure 1B). Interestingly, although the stable targeting of NrCAM to the AIS was dependent on Nfasc186, the converse was not true (see Figure S1 available online); neither was NrCAM required for the long-term stability of the AIS (Figure S1). We concluded that although Nfasc186 is not required for in vivo assembly of voltage-gated sodium channels at the AIS, it recruits NrCAM to the AIS complex.

### Nfasc186 Stabilizes the AIS

**Figure 2:**
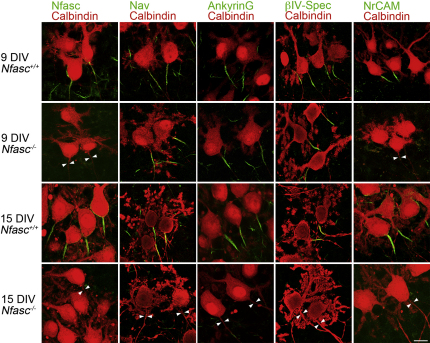
*Nfasc* Is Required to Maintain the AIS Complex In Vitro Immunofluorescence of Calbindin-positive Purkinje cells in organotypic cerebellar slices from newborn *Nfasc^−/−^* and wild-type littermates. At 9 days in vitro (DIV), AIS proteins with the exception of NrCAM are correctly localized in wild-type slices. However, by 15 DIV Na_v_, AnkyrinG, and βIV-spectrin are no longer localized to the AIS in mutant slices (arrowheads point to AIS location). Scale bar, 10 μm.

Since the Neurofascins are not required for the clustering of sodium channels or the majority of their associated proteins in the AIS complex, we asked if instead they have a role in maintaining the complex. Since Neurofascin null mice die at P7 (Sherman et al., 2005), it is not possible to study the long-term stability of their initial segments in vivo. Hence, we first examined organotypic slice cultures derived from Neurofascin null cerebella. Such cultures are known to maintain viability for months (Kessler et al., 2008). In the absence of the Neurofascins clustering of components of the AIS was complete after 9 days in vitro (DIV). The exception was NrCAM, as found in vivo (Figures 1A and 2). Further culture for up to 15 days resulted in the dispersal of sodium channels, AnkyrinG and βIV-Spectrin, whereas the wild-type AIS remained intact (Figure 2). This suggests that the Neurofascins are required for AIS stability, at least in vitro.

To determine if the neuronal isoform of Neurofascin, Nfasc186, is also required for AIS stability in vivo, we used an inducible conditional knockout approach to inactivate the *Nfasc* gene specifically in adult neurons. To achieve this we generated mice carrying a floxed allele of Neurofascin (see Experimental Procedures) and a transgenic line in which the CreERT2 cassette was driven by the Thy1.2 promoter (TCE) (Caroni, 1997; Feil et al., 1997). Using a reporter line, we showed that these TCE mice expressed tamoxifen-inducible Cre robustly in cerebellar Purkinje cells (Figure S2).

**Figure 3:**
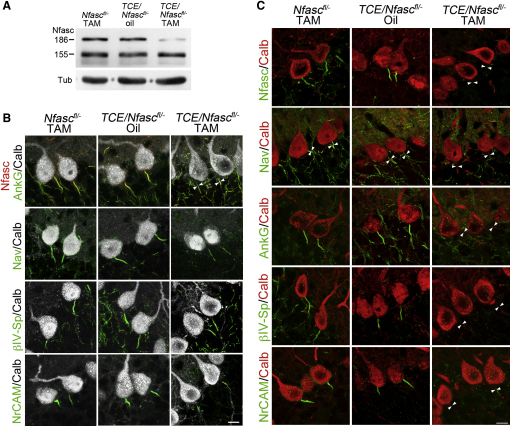
Nfasc186 Is Required to Maintain the AIS In Vivo (A) Western blotting using a pan-anti-Neurofascin antibody (NFC1) of hindbrain homogenates (20 μg per lane) from *TCE/Nfasc^fl/−^* mice 6 weeks after tamoxifen (TAM) treatment showed that the levels of the Nfasc186 neuronal isoform are significantly reduced whereas the Nfasc155 glial isoform is unaffected. Controls comprised *Nfasc^fl/−^* and *TCE/Nfasc^fl/−^* mice treated with tamoxifen and sunflower oil (carrier solution) respectively. Tubulin was the loading control. (B) Immunofluorescence of Calbindin positive Purkinje cells in cryostat sections of cerebella from *Nfasc^fl/−^* and *TCE/Nfasc^fl/−^* mice 3 weeks after tamoxifen (TAM) treatment shows that although Nfasc186 is lost, other components of the AIS, including sodium channels, are still present. Controls comprised *Nfasc^fl/−^* and *TCE/Nfasc^fl/−^* mice treated with tamoxifen and sunflower oil (carrier solution) respectively. (C) Immunofluorescence of Calbindin positive Purkinje cells in cryostat sections of cerebella from *Nfasc^fl/−^* and *TCE/Nfasc^fl/−^* mice 6 weeks after tamoxifen (TAM) treatment shows that Nfasc186 is required to maintain the integrity of the AIS complex (arrowheads point to AIS location). Controls as for (B). See also Figure S2.

To inactivate the *Nfasc* gene efficiently using tamoxifen induction of Cre activity, we generated *TCE* transgenic mice with one floxed and one null allele of the gene (*TCE/Nfasc^fl/−^*). Western blot analysis of hindbrain homogenates from *TCE/Nfasc^fl/−^* mice 6 weeks after tamoxifen treatment showed that recombination resulted in a reduction in the level of Nfasc186, whereas the glial isoform (Nfasc155) was unaffected (Figure 3A). Although we focused our analysis on brains 6 weeks posttamoxifen to ensure complete loss of Nfasc186 at AIS and AIS disruption, the disappearance of Nfasc186 at the AIS was clear at 3 weeks after tamoxifen-induced recombination, a time when the other components of the complex were still present (Figure 3B). Although there was some reduction in the length of NrCAM staining at 3 weeks, it was not lost completely until 4 weeks posttamoxifen. Between 3 and 4 weeks posttamoxifen, the kinetics of AnkyrinG, βIV-Spectrin, and NrCAM loss in vivo were rapid and coincident with the disappearance of sodium channel immunostaining at the AIS, which was complete by 4 weeks, thus precluding an informative evaluation of the sequence in which these components are lost (data not shown). Nfasc186 was efficiently eliminated at the AIS of Purkinje cells 6 weeks posttamoxifen (Figure 3C), since the number of Purkinje cells immunopositive for Nfasc186 was reduced from 99.2% ± 0.8% to 2.5% ± 2.5% (mean values ± SEM, n = 3, 40 cells per animal, p < 0.0001, unpaired Student's t test). Furthermore, and consistent with the results of the cerebellar slice culture experiment with Neurofascin null mice (Figure 2), loss of Nfasc186 from the AIS abolished the immunofluorescence signal for sodium channels, AnkyrinG, βIV-Spectrin, and NrCAM (Figure 3C). No demyelination was observed and the levels of myelin proteins, as assessed by western blotting, were unchanged (data not shown).

Together, these in vitro and in vivo data suggest a distinct role for Nfasc186 in maintaining the mature configuration of the AIS. Thus, whereas assembly of the AIS appears to involve AnkyrinG acting as a master coordinator (Dzhashiashvili et al., 2007; Sobotzik et al., 2009) and does not require Nfasc186, maintenance of the AIS, including AnkyrinG localization, appears to require Nfasc186. Because Nfasc186 is also believed to be important for the establishment of inhibitory synaptic input from basket cells onto Purkinje cells (Ango et al., 2004) and because an intact AIS is thought to be required for initiation of spike output from Purkinje cells (Khaliq and Raman, 2006), we next asked if loss of the AIS following deletion of Nfasc186 affects these key functional attributes of the AIS.

### Disruption of Pinceau Synapses following Deletion of Nfasc186

**Figure 4:**
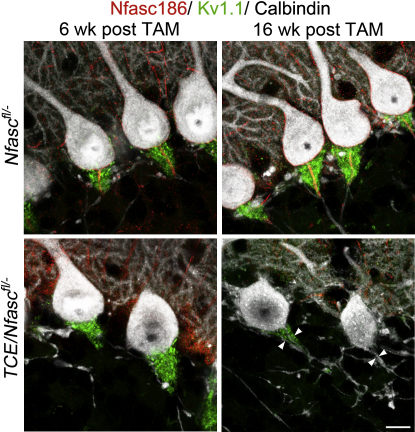
Pinceau Synapses with Purkinje Cells Are Unstable without an Intact AIS Immunofluorescence of Purkinje cells in vibratome sections of cerebella from *Nfasc^fl/−^* and *TCE/Nfasc^fl/−^* mice 6 and 16 weeks after tamoxifen (TAM). In the absence of an intact AIS pinceau synapses labeled with antibodies against K_v_1.1 are still intact at 6 weeks posttamoxifen. However, by 16 weeks they are either disrupted or absent (arrowheads point to AIS location). Scale bar, 10 μm.

Pinceau synapses are formed by basket cell terminals which provide GABAergic input to Purkinje cells at the AIS. Previous studies have implicated Nfasc186 and AnkyrinG in ensuring appropriate targeting of these synaptic inputs to the Purkinje cell AIS (Ango et al., 2004; Huang, 2006). Therefore, we were interested to determine if an intact AIS is required to maintain pinceau synapses. Figure 4 shows that it took considerably longer for these structures to disassemble than the AIS. Thus, 6 weeks after tamoxifen, when the AIS is severely disrupted, the adjacent pinceau synapses remain, whereas 16 weeks after tamoxifen the pinceau synapses are absent or substantially reduced in size. All were affected. Therefore, once pinceau synapses are assembled around the AIS, neither Nfasc186 nor its colocalized proteins in the AIS appear to be directly responsible for their maintenance.

### Prolonged Stability of Nfasc186 at Nodes of Ranvier

**Figure 5:**
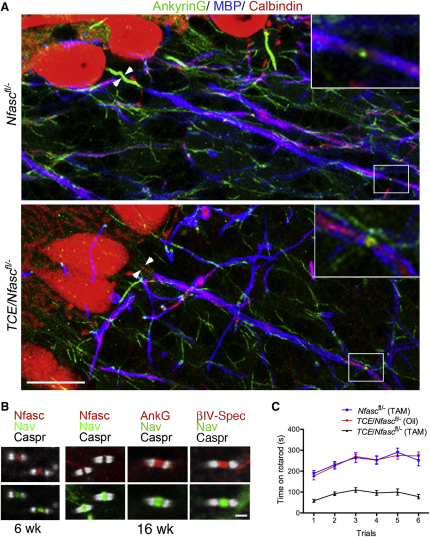
Conditional Deletion of Nfasc186 Disrupts the AIS but Leaves Nodes Intact (A) Immunofluorescence of parasagittal vibratome sections of cerebella from *Nfasc^fl/−^* and *TCE/Nfasc^fl/−^* mice 6 weeks after tamoxifen showing Calbindin positive Purkinje cells and their axons. An antibody versus the myelin marker, myelin basic protein (MBP in blue), was used to identify the position of the first node of Ranvier (insets). The AIS (arrowheads) and nodes were labeled using an AnkyrinG antibody. AnkyrinG is localized at the AIS of *Nfasc^fl/−^* Purkinje cells but not in *TCE/Nfasc^fl/-^* cerebella. Nevertheless nodes in both mice (insets) are still AnkyrinG positive. Scale bar, 20 μm. (B) Immunofluorescence analysis of cerebellar white matter tracts from *TCE/Nfasc^fl/−^* mouse after tamoxifen treatment shows that both Nfasc186 and voltage-gated sodium channels (Na_v_) are initially clustered at nodes of Ranvier at 6 weeks posttamoxifen but that Nfasc186 is severely depleted by 16 weeks. Nevertheless, the localization of Na_v,_ AnkyrinG, and βIV-spectrin at the nodes is preserved. Nodes were located by immunostaining for the paranodal marker Caspr. Scale bar, 2 μm. (C) Three groups of mice (10 mice per group) were tested 6 weeks after tamoxifen. The means ± SEM for time spent on the rotarod across six trials are shown for *Nfasc^fl/−^* mouse treated with tamoxifen (blue), *TCE/Nfasc^fl/−^* mice treated with sunflower oil (red), and *TCE/Nfasc^fl/−^* mice treated with tamoxifen (black). There was no significant difference in performance between the two control groups (*Nfasc^fl/−^* mouse treated with tamoxifen versus *TCE/Nfasc^fl/−^* mice treated with sunflower oil; p > 0.05). However, the differences observed between each of the control groups and the *TCE/Nfasc^fl/−^* mouse treated with tamoxifen were highly significant (p < 0.001). See also Movie S1. Error bars indicated standard error of the mean.

Since we have previously found that Nfasc186 has a role in the assembly of nodes of Ranvier in vivo (Sherman et al., 2005; Zonta et al., 2008), we wondered if the inducible deletion of Nfasc186 from mature animals would also affect nodes of Ranvier. We found that CNS nodes of Ranvier remained intact at a time when the Purkinje cell AIS was disrupted (Figures 5A and 5B). Nfasc186 was lost from nodes of Ranvier between 6 and 16 weeks after tamoxifen-induced recombination (from 97% ± 2.1% to 19% ± 1.4%; mean values ± SEM, n = 3, 40 nodes per animal) (Figure 5B). The fact that Nfasc186 persisted at nodes of Ranvier in myelinated CNS axons even 6 weeks after tamoxifen treatment may have contributed to the resistance of nodes to disruption (Figure 5B). Nevertheless, from 6 weeks to 16 weeks sodium channels flanked by the paranodal axoglial junction marker Caspr persisted at CNS nodes of Ranvier even in the absence of Nfasc186 (from 98% ± 0.8% to 90% ± 1.3%; mean values ± SEM, n = 3, 40 nodes per animal) (Bhat et al., 2001; Sherman et al., 2005; Zonta et al., 2008; Figure 5B).

### Mature Expression of Nfasc186 Is Required for Normal Motor Behavior and Action Potential Firing by Cerebellar Purkinje Cells

To address the functional consequences of disassembling the AIS, we evaluated motor behavior and Purkinje cell action potential firing at 6 weeks posttamoxifen. We chose this time point as the AIS is disrupted, but pinceau synapses and nodes of Ranvier remain intact.

Mutants had an altered gait (Movie S1), and testing their motor coordination and balance using a rotarod revealed significant deficits in mutant animals compared to controls (Figure 5C). Clearly this cannot be attributed solely to disruption of Purkinje cell AIS function since many other neuronal cell types including spinal motor neurons are affected (data not shown). Nevertheless, it was perhaps surprising to observe the relative mild nature of the phenotype in animals behaving in their normal activities. This prompted us to ask how essential the intactness of the initial segment was for electrophysiological function.

**Figure 6:**
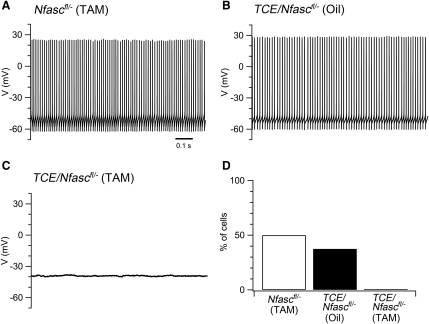
Disassembly of the AIS Disrupts Spontaneous Action Potential Firing by Purkinje Cells (A and B) Examples of spontaneous action potentials generated by Purkinje cells from control *Nfasc^fl/−^* (TAM) and *Nfasc^fl/−^* (Oil) mice. (C) Example of resting membrane potential of a Purkinje cells from a *TCE/Nfasc^fl/−^* (TAM) mouse. (D) Percentage of Purkinje cells able to maintain spontaneous firing for >10 min in the *Nfasc^fl/−^* (TAM), *Nfasc^fl/−^* (Oil), and *TCE/Nfasc^fl/−^* (TAM) mice (n = 32, 26, 24 Purkinje cells, respectively; 4–5 mice per group; χ^2^_(2,n = 82)_ = 14.98, p < 0.005). See also Figure S3.

**Figure 7:**
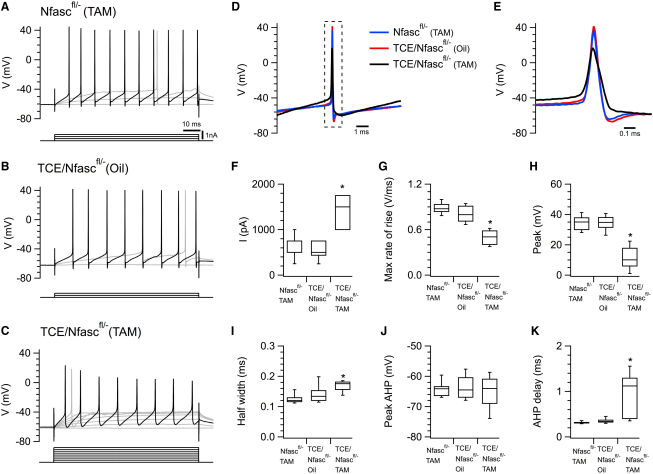
An Intact AIS Is Not Necessary for Action Potential Firing but Affects Action Potential Properties (A–C) Examples of membrane potential responses to positive current steps recorded from Purkinje cells from *Nfasc^fl/−^* (TAM) (A), *TCE/Nfasc^fl/−^* (Oil) (B), and *TCE/Nfasc^fl/−^* (TAM) mice (C). The membrane potential was adjusted to −60 mV by injection of a negative holding current. Highlighted traces illustrate responses for which action potentials frequencies are in the 52–58 Hz range chosen for analysis. The frequency of these action potentials did not differ between groups (*Nfasc^fl/−^* (TAM)52 ± 3.66, TCE/*Nfasc^fl/−^* Oil 57.78 ± 3.30, TCE/*Nfasc^fl/−^* (TAM) 58.09 ± 2.8 Hz, F_2,18_ = 0.94, p > 0.05). (D) Expanded and superimposed waveforms of the second spikes in the highlighted traces in (A)–(C). (E) Expanded spikes from the region indicated by the box in (D). (F) The current required to drive action potentials at a frequency of 52–58 Hz was greater in *TCE/Nfasc^fl/−^* (TAM) mice (n = 7) compared with either *Nfasc^fl/−^* (TAM) (n = 8) or *TCE/Nfasc^fl/−^* (Oil) (n = 6) (3 mice per group; F_2,18_ = 22.84, p < 0.0001). (G) The maximum rate of rise of the action potential was decreased in *TCE/Nfasc^fl/−^* (TAM) mice compared with both *Nfasc^fl/−^* (TAM) or *TCE/Nfasc^fl/−^* (Oil) (F_2,18_ = 39.75, p < 0.0001). (H) The peak action potential depolarization was reduced in *TCE/Nfasc^fl/-^* (TAM) mice compared with both *Nfasc^fl/−^* (TAM) or *TCE/Nfasc^fl/−^* (Oil) (F_2,18_ = 39.86, p < 0.0001). (I) The half-width of the action potential was increased in *TCE/Nfasc^fl/−^* (TAM) mice compared with both *Nfasc^fl/−^* (TAM) or *TCE/Nfasc^fl/−^* (Oil) (F_2,18_ = 8.34, p = 0.003). (J) There was no difference between groups in the peak of the afterhyperpolarization (F_2,18_ = 0.35, p = 0.71). (K) The delay until the peak of the afterhyperpolarization was increased in *TCE/Nfasc^fl/−^* (TAM) mice compared with both *Nfasc^fl/−^* (TAM) or *TCE/Nfasc^fl/−^* (Oil) (F_2,18_ = 15.28, p < 0.0001). ^∗^p < 0.05 versus *Nfasc^fl/−^* (TAM) and *TCE/Nfasc^fl/−^* (Oil), Fisher's PLSD. In the box plots, the horizontal bar is the median, the boxes indicate the 25th–75th percentile range, and the vertical lines indicate the 10th–90th percentile range.

Purkinje cells in awake animals typically maintain spontaneous action potential firing indefinitely (Armstrong et al., 1979; Schonewille et al., 2006; Thach, 1968); hence, in previous work in vitro we selected for analysis Purkinje cells that generate stable and continuous action potential firing (Nolan et al., 2003). In this study, we find that 37%–50% of Purkinje cells from control *Nfasc^fl/−^* (TAM) and *TCE/Nfasc^fl/−^* (Oil) mice maintained spontaneous action potential firing for > 10 min (Figures 6A, 6B, and 6D). The properties of these spontaneous action potentials did not differ between the two control groups and were similar to spontaneous action potentials recorded previously (Häusser and Clark, 1997; Nolan et al., 2003), indicating that tamoxifen and the expression of Cre do not on their own affect spike firing (Figure S3). In contrast to the control groups, none of the Purkinje cells in which the initial segment was disassembled were able to maintain spontaneous action potential firing for > 10 min (χ^2^_(2, n = 82)_ = 14.98, p < 0.005) (Figures 6C and 6D). Instead, these neurons maintained a modal resting potential of −43.0 ± 1.8 mV (n = 19) (Figure 6). Using less restrictive criteria, in which we simply compared the number of cells that could generate spontaneous action potentials at any point during a recording, we found that a majority of Purkinje cells from *Nfasc^fl/−^* (TAM) (73.08%, 19/26), and *TCE/Nfasc^fl/−^* (Oil) groups (68.75%, 22/32) fired action potentials spontaneously, whereas spontaneous action potential firing was rarely observed in Purkinje cells obtained from *TCE/Nfasc^fl/−^* (TAM) mice (8.33%, 2/24). Nevertheless, Purkinje cells from all *TCE/Nfasc^fl/−^* (TAM) mice were able to generate action potentials in response to positive current steps (Figure 7C), indicating that an intact initial segment is not required for evoked action potential firing.

We next asked if disassembly of the initial segment alters the properties of evoked action potentials. For these experiments, the membrane potential was adjusted to −60 mV by injection of a negative holding current and action potentials were evoked by superimposed positive current steps (Figures 7A–7E). The current required to drive spike firing at a frequency of 52–58 Hz was significantly greater in *TCE/Nfasc^fl/−^* (TAM) mice compared with either control group (Figure 7F). Comparison of the waveform of action potentials evoked at this frequency revealed that deletion of Nfasc186 leads to a reduced maximum rate of rise (Figure 7G) and peak amplitude (Figure 7H) and increased half-width (Figure 7I), but no difference in the peak membrane potential of the afterhyperpolarization (AHP) (Figure 7J) although there was an increase in the delay until the peak of the AHP (Figure 7K). There was no significant difference between groups in the frequency of spikes chosen for analysis (F_2,18_ = 0.94, p = 0.41) or in the input resistance of Purkinje cells estimated from their response to negative current steps (F_2,17_ = 1.93, p = 0.18). There was also no significant difference in the voltage threshold for spike firing (F_2,18_ = 1.42, p = 0.27), although because of cell-to-cell variability in the voltage threshold, detection of expected differences might require substantially larger data sets. The differences in action potential properties that we find between groups are consistent with initiation of action potentials in control neurons taking place at the AIS (Khaliq and Raman, 2006; Palmer et al., 2010). Slower and smaller action potentials observed following disassembly of the AIS suggest that action potentials can also initiate from more distal locations, presumably the first node of Ranvier (Clark et al., 2005).

Together, these results validate the molecular disintegration of the axon initial segment and underline the essential nature of Nfasc186 to AIS function and stability. They support the idea that spontaneous action potential firing by cerebellar Purkinje cells relies on an intact molecular configuration of the AIS. Nevertheless, they suggest that an intact initial segment is not necessary for action potential firing, but that it is a critical determinant of the threshold and waveform of action potentials generated by cerebellar Purkinje cells.

## Discussion

The selective loss of the neuronal isoform of Neurofascin, Nfasc186, at the AIS together with the preservation of intact nodes of Ranvier and pinceau synapses has allowed us to address two major questions. First, is Nfasc186 necessary for the assembly or stabilization of the AIS, and second, what is the role of the initial segment in mature neurons? We show that while not required for AIS assembly, Nfasc186 is essential for AIS stabilization. Ablation of Nfasc186 leads to disintegration of the AIS complex and the loss of key components including voltage-gated sodium channels. Furthermore, perturbing the molecular composition of the AIS leads to the longer-term loss of pinceau synapses, but localization of these synapses does not appear to require an intact AIS in the short-term. Purkinje cells with a disrupted AIS, but intact nodes of Ranvier are no longer able to fire spontaneous spikes. Nevertheless, following stimulation they are able to generate evoked action potentials, albeit with significantly altered characteristics. Together, these data suggest that in the mature nervous system Nfasc186 maintains normal action potential initiation by stabilizing the AIS. We suggest a model for stabilization of the mature AIS that follows a molecular logic distinct from its assembly, but with similarities to the assembly and stabilization of nodes of Ranvier. Thus, whereas distinct mechanisms are required for the cell-autonomous formation of the AIS compared with the clustering of nodal proteins dependent on axo-glial interaction, once the AIS is formed the important role for Nfasc186 at the nodes is recapitulated in the AIS complex (Sherman et al., 2005; Zonta et al., 2008). The fact that sodium channels persist at nodes after Nfasc186 has been eliminated likely reflects the known importance of the paranodal axoglial junction in clustering the nodal complex in the CNS (Zonta et al., 2008).

### Nfasc186 Has Distinct Roles in Assembly and Maintenance of the AIS

There is convincing evidence that AnkyrinG has a key developmental role in AIS assembly during the clustering of key components of the initial segment, namely voltage-gated sodium channels, Nfasc186, βIV-Spectrin, and NrCAM (Dzhashiashvili et al., 2007; Jenkins and Bennett, 2001; Zhou et al., 1998). Furthermore, studies of cultured hippocampal neurons have indicated that AIS assembly is independent of Nfasc186 and that Nfasc186 is recruited to this domain via its interactions with AnkyrinG (Dzhashiashvili et al., 2007). In long-term cultures of such neurons loss of AnkyrinG led to the derangement of preformed initial segments (Hedstrom et al., 2008). And there is evidence both in vitro and in vivo that loss of AnkyrinG from the AIS can induce a concomitant loss of neuronal polarity (Hedstrom et al., 2008; Rasband, 2010; Sobotzik et al., 2009). Our data confirm the view that Nfasc186 is not critical for AIS assembly during development. In contrast, we show that in adult animals Nfac186 is absolutely required for the maintenance of the integrity of this domain. The other L1 family member at the AIS, NrCAM, is recruited through its interaction with Nfasc186 but is required neither for the clustering nor the stabilization of sodium channels at the AIS.

How might Nfasc186 become indispensable for AIS structure and function after the other molecular components of the complex have been assembled? During development Nfasc186 is presumed to be recruited to the AIS through its interactions with AnkyrinG, but the latter can also interact with sodium channels, NrCAM, and βIV-spectrin (Davis and Bennett, 1994; Dzhashiashvili et al., 2007; Garrido et al., 2003; Jenkins and Bennett, 2001; Komada and Soriano, 2002). However, a key feature of Nfasc186, by comparison with AnkyrinG, is that it is potentially able to act as a linker between proteins located inside the neuron, such as AnkyrinG itself, and extracellular proteins such as Brevican (Rasband, 2010). Although NrCAM could, in principle, have a similar role, it seems to function primarily as an ancilliary interactor of Nfasc186. Further, once recruited to the AIS Nfasc186 can also interact with the beta subunits of sodium channels (Ratcliffe et al., 2001). The ability of Nfasc186 to link key extracellular and membrane components may be critical to its role in stabilization of the AIS in adult neurons.

Based on these data, we propose a model for stabilization of the mature AIS complex in which Nfasc186 has a function similar to its role at the node of Ranvier. According to this model, in the mature AIS Nfasc186 acts as an anchor for recruitment of new proteins to replenish molecules removed for degradation. Whereas initial localization and assembly of the AIS relies on AnkyrinG, our stabilization model predicts that interactions of the extracellular domain of Nfasc186 with components of the extracellular matrix, for example proteins such as Brevican (Rasband, 2010), or directly with cells that surround the AIS, maintain the localization of the AIS complex. The intracellular domain of Nfasc186-NrCAM is then used to anchor AnkyrinG and other components of the AIS complex through interactions similar to those used to assemble nodes of Ranvier (Rasband, 2010; Sherman and Brophy, 2005). Hence, according to this model, loss of Nfasc186 will lead to instability of sodium channels and concomitant delocalization of their associated AnkyrinG and NrCAM. Our model does not rule out the possibility that AnkryinG is also required for maintenance of the AIS by stabilizing Nfasc186, similar to its role at nodes of Ranvier (Dzhashiashvili et al., 2007). From the current study, it was not possible to differentiate the sequence of AIS component disassembly following Nfasc186 loss. Similarly, two different studies on the AIS of cultured neurons have found that the simultaneous accumulation of Nav channels, AnkyrinG, βIV-Spectrin, NrCAM, and Neurofascin did not permit a differential analysis of the assembly of individual AIS components (Boiko et al., 2007; Hedstrom et al., 2008).

An intriguing consequence of inactivating the *Nfasc* gene in adult neurons was the longer persistence of Nfasc186 at nodes of Ranvier in contrast to the AIS. This suggests that Nfasc186 has a shorter half-life at the AIS compared to nodes. According to the model we propose above, this difference would be expected if the major difference between the mature AIS and nodes of Ranvier is the rate of turnover of their constituent molecules. This is consistent with the emerging view that plasticity of the AIS may play a role in modulating the electrical properties of neurons (Grubb and Burrone, 2010a, 2010b). The enhanced sensitivity of the AIS to hypoperfusion-induced hypoxia (Schafer et al., 2009) may also reflect the fact that the AIS is inherently less stable than related structures, such as nodes. The fundamental role we propose for Nfasc186 in anchoring new proteins may represent an important target in regulating normal AIS function.

The formation of pinceau synapses between basket cell axons and the AIS of Purkinje cells in the cerebellum has been shown to be disrupted either in the absence of AnkyrinG or by using a dominant-negative form of Nfasc186 (Ango et al., 2004). Here, we have shown that the intact AIS is also essential for maintenance of pinceau synapses. However, the persistence of apparently intact pinceau synapses for some time after AIS disruption indicates a role for other proteins in contributing to the stabilization of these structures. The perineuronal nets formed by the extracellular matrix are possible candidates (Celio et al., 1998; Rasband, 2010).

### Maintenance of the AIS by Nfasc186 Is Critical for Normal Action Potential Initiation

Profound differences in action potential firing by Purkinje cells following deletion of Nfasc186 are consistent with our anatomical observation of AIS disintegration. Previous studies on the role of the AIS have relied on pharmacological manipulation of AIS function (Khaliq and Raman, 2006; Palmer et al., 2010). These have led to the view that action potentials initiate from the AIS; however, the advantages for neuronal function conferred by spatially restricting initiation of the action potentials are not well understood. Our data suggest that disruption of the AIS prevents generation of spontaneous spikes by Purkinje cells and that it modifies the waveform of evoked action potentials. These data support the idea that the AIS plays a critical role in spike initiation in mammalian neurons. However, while our results do not rule out changes that might in part enable Purkinje cells to adapt to disruption of their AIS, they nevertheless suggest that an intact AIS is not necessary for generation of somatic action potentials, but rather that it promotes normal function by modifying action potential initiation. In Purkinje cells, this may be important for generation of spontaneous action potentials at relatively high frequencies, whereas in other neuron types it may contribute to establishing rules for somatodendritic integration of synaptic inputs (Mainen et al., 1995; Stuart and Sakmann, 1994). This modulatory function is consistent with the absence of an AIS in invertebrate neurons, which nonetheless rely on generation of action potentials (Rasband, 2010).

Action potentials generated by Purkinje cells are the sole output from the cerebellar cortex and synaptic input to Purkinje cells modifies the frequency of intrinsically generated spontaneous action potentials (Häusser and Clark, 1997; Raman and Bean, 1997). Pharmacological block of Na_v_ channels at the initial segment abolishes spontaneous firing of action potentials by cerebellar Purkinje cells and attenuates evoked action potentials (Khaliq and Raman, 2006). However, other studies have implicated both the AIS and the first node as critical in action potential generation in Purkinje cells (Clark et al., 2005; Palmer et al., 2010). The absence of spontaneous action potentials following disturbance of the AIS is consistent with the notion that spontaneous action potentials initiate from the AIS (Fleidervish et al., 2010; Khaliq and Raman, 2006; Palmer et al., 2010). In contrast, the ability to evoke action potentials in the absence of an AIS suggests that at least in principle the AIS is not required for action potential initiation, although in physiological conditions the lower threshold conferred by the AIS is likely to cause it to be the main site of spike initiation in Purkinje cells (Fleidervish et al., 2010; Khaliq and Raman, 2006; Palmer et al., 2010).

The susceptibility of the initial segment to disruption that we have demonstrated here has implications for human disease. Neurofascin has been identified as a prime target in the autoimmune attack of axons associated with multiple sclerosis (Mathey et al., 2007). And the results presented here predict that this autoimmune response will selectively destabilize the AIS. Further, it has been shown in a stroke model that AIS are much more susceptible to hypoxia-induced proteolytic degradation than nodes of Ranvier (Schafer et al., 2009). Hence, irrespective of the initial insult, the vulnerability of the AIS to attack is likely to undermine neuronal function.

In summary, we find that following assembly of the AIS, Nfasc186 appears to act as an anchor that maintains the appropriate localization of critical components including AnkryinG and sodium channels. Modified action potential firing following deletion of Nfasc186 is consistent with these anatomical observations, while also supporting the view that, although an intact AIS is not necessary for action potential initiation, it modulates action potential firing. Together our results suggest that distinct molecular mechanisms are used for the developmental assembly and the adult maintenance of the AIS. This may be critical for flexible regulation of computations that transform synaptic input into patterns of spike output suitable for the control of downstream neurons.

## Experimental Procedures

### Animals

All animal work conformed to UK legislation (Scientific Procedures) Act 1986, and to the University of Edinburgh Ethical Review Committee policy. The generation of *Nfasc*^−/−^ mice has been described (Sherman et al., 2005). *Nfasc^flox^* mice were generated following the same strategy, but with an alternative excision where only the PGK*neo*-HSV*tk* cassette was removed and where the preserved exon 4 was flanked by two *loxP* sites. Transgenic mice expressing a full-length cDNA encoding Nfasc186 or a cDNA encoding the inducible Cre recombinase CreERT2 under the control of the *Thy1.2* promoter (Caroni, 1997) were generated by pronuclear injection as described (Sherman and Brophy, 2000). For the *Thy1Nfasc186* construct, a FLAG tag sequence was first inserted at the 3′ of the coding region. The cDNA was then cloned into the XhoI site of the pTSC21k vector (Lüthi et al., 1997) and was released using NotI. After backcrossing to a C57BL/6 background, one of the lines was interbred with *Nfasc^+/−^* mice to generate *Nfasc^−/−^/Nfasc186* mice. The *Thy1CreERT2* transgene comprised cDNA encoding CreERT2 excised from the pCreERT2 vector (Feil et al., 1997; Imai et al., 2001) using EcoRI after which it was blunt ended, cloned into the XhoI site of the pTSC21k vector, and released using NotI. After backcrossing to a C57BL/6 background, the *Thy1CreERT2* (TCE) line was interbred with the *Rosa26-YFP* (Srinivas et al., 2001) reporter line or successively interbred with *Nfasc^+/−^* and *Nfasc^fl/fl^* mice to generate *Nfasc^−/fl^/Thy1CreERT2* mice.

### Induction of Recombination with Tamoxifen

Tamoxifen (Sigma) was dissolved in sunflower oil/ethanol (10:1 ratio) at 10 mg/ml. Recombination was induced by intraperitoneal injection of 0.18 mg/g body weight/day into 3-week-old animals for 5 consecutive days. Control *Nfasc^−/fl^/Thy1CreERT2* were treated with the carrier solution (sunflower oil/ethanol 10:1). This injection protocol was repeated 10 days later. Mice were sacrificed 6 or 16 weeks after the second treatment.

### Organotypic Cerebellar Slices

Brains from newborn *Nfasc*^−/−^ and control mice were dissected into ice-cold Hank's Balanced Salt Solution (HBSS; Sigma) to remove meninges and forebrain. Parasagittal cerebellar slices (250 μm) were cut using a McIlwain tissue chopper and separated in culture medium composed of 50% Minimum Essential Medium Eagle (MEM, Sigma), 25% Earle's Balanced Salt Solution (Sigma), 25% heat-inactivated horse serum (Sigma), glucose (6.5 mg/ml), L-glutamine (2 mM), penicillin-streptavidin solution (100 mg/ml) (Sigma), and Amphotericin B solution (Sigma). The slices were transferred to the membrane of 30 mm culture inserts (Millicell, Millipore) with prewarmed medium and were maintained in a 37% incubator with 5% CO_2_ enriched humidified atmosphere. Culture medium without Amphotericin B was replaced on the day after slice preparation and changed every 2 days.

### Antibodies, Microscopy, and Western Blots

For immunostaining of organotypic cerebellar preparation, the slices cultured 9 DIV or 15 DIV were fixed by immersion in 4% paraformaldehyde in 0.1 M sodium phosphate buffer (pH 7.4) for 1 hr at room temperature, followed by washes in PBS. Pieces of membrane containing single or multiple slices were cut out and immunostaining was performed in 6-well tissue culture plates.

Immunostaining of 10–12 μm cerebellum sections was performed after transcardial perfusion with 4% paraformaldehyde, 0.1 M sodium phosphate buffer (pH 7.4) as described previously (Tait et al., 2000). For vibratome sections, the brains were postfixed with 4% paraformaldehyde, 0.1 M sodium phosphate buffer (pH 7.4) overnight before being washed in several changes of 0.1 M phosphate buffer and cut in 50 μm parasagittal sections using an Intracell 1000 vibratome. Goat anti-Kv1.1 (1:100, Santa-Cruz); mouse anti-Calbindin (1:1000, Sigma); mouse anti-AnkyrinG IgG2a, clone N106/36 (1:50, Neuromab); rabbit anti-Calbindin (1:5000, Swant); and rabbit anti-GFP (1:500, Invitrogen) were used at the indicated dilutions. Rabbit anti-Nav (1:200) was generated after immunization with the synthetic peptide TEEQKKYYNAMKKLGSKKPK with an N-terminal cysteine conjugated to KLH. The peptide sequence corresponds to the intracellular III-IV loop of Nav channels and is identical in all known vertebrate Nav channels (Catterall, 1995). All other primary and secondary antibodies have been described (Sherman et al., 2005; Tait et al., 2000; Zonta et al., 2008). Cerebellar slices used for electrophysiology were subsequently stained by floating immunohistochemistry with rabbit MNF2 (1:100) (Tait et al., 2000) specific for Nfasc186 and mouse anti-calbindin (1:500) in 10% fish gelatin, Triton 0.5% in PBS) incubated overnight followed by Cy3-conjugated donkey anti-rabbit (1:600) and goat AlexaFluor 647-conjugated anti-mouse IgG1 (1:200). For confocal microscopy, we used a Leica TCL-SL confocal microscope with either a 20× or a 63× objective, 1.4 NA, and Leica proprietary software. The acquired stacks were assembled using the maximum projection tool. All figures were prepared using Adobe Photoshop CS4 extended version 11. Western blotting was performed as described (Sherman et al., 2005) on hindbrain lysates (20 μg protein per lane). The blot shown in Figure 3A was replicated in three different preparations.

### Rotarod

Mice (10 per group, equal number of males and females) were tested 6 weeks after tamoxifen treatment by two trials per day for 3 consecutive days using a Ugo Basile rotarod with an accelerating rotation speed from 4 to 40 rotations/min in 300 s. Each trial comprised three experiments separated by 15 min of rest. The latency to fall for each of the three experiments was recorded and subsequently averaged. Statistical analysis was by two-way ANOVA and t tests with GraphPad Prism 5.0c software.

### Electrophysiology

Whole-cell patch-clamp recordings were made from Purkinje cells in parasagittal brain slices obtained from 12- to 14-week-old mice as previously described (Nolan et al., 2003). Briefly, slices of thickness 200 μm containing the cerebellar vermis were sectioned using a Vibratome 3000. For sectioning, brains were submerged under cold (4°C–6°C) oxygenated modified artificial cerebrospinal fluid (ACSF) of the following composition (mM): NaCl 86, NaH_2_PO_4_ 1.2, KCl 2.5, NaHCO3 25, CaCl_2_ 0.5, MgCl_2_ 7, glucose 25, sucrose 75. Slices were then maintained in oxygenated standard ACSF (mM): NaCl 124, NaH_2_PO_4_ 1.2, KCl 2.5, NaHCO_3_ 25, CaCl_2_ 2, MgCl_2_ 1, glucose 20. Immediately following sectioning slices were maintained at 37°C ± 1°C for 10–20 min and subsequently at room temperature for a minimum of 40 min. For recording, slices were visualized under a microscope with infrared illumination while being maintained in oxygenated standard ACSF at 37°C ± 1°C.

Recording electrodes were filled with intracellular solution of the following composition (mM): Kgluconate 130, KCl 10, HEPES 10, MgCl_2_ 2, EGTA 0.1, Na_2_ATP 2, Na_2_GTP 0.3, NaPhosphocreatine 10, and biocytin 2.7. The electrode resistance in the bath containing standard ACSF was 3–5 MΩ. Current-clamp recordings were made with a Multiclamp 700A amplifier (Molecular Devices), sampled at 50 KHz and filtered at 10 KHz. Appropriate bridge and electrode capacitance compensation were applied. Cells with series resistance >25 MΩ were excluded. An experimentally measured liquid junction potential of +8.1 mV (bath potential relative to the patch-pipette) for the standard ACSF was not corrected for.

Data were analyzed using custom written routines in IGOR pro (Wavemetrics). Statistical analysis was performed in Statview using Student's t test, chi-square test, or one-way ANOVA followed by Fisher's PLSD post hoc when allowed. Level of significance was set at <0.05.

## Supplemental Information

Document S1. Three Figures

Movie S1. Mice with a Disrupted AIS Have Altered Gait At the beginning of the movie, the mouse on the left is a control littermate for the mouse on the right in which inactivation of the Neurofascin gene in neurons had been initiated 6 weeks earlier by tamoxifen administration. See also Figure 5.
