# Supplementary material for: A Critical Role for Neurofascin in Regulating Action Potential Initiation through Maintenance of the Axon Initial Segment
Source: Neuron. 2011 Mar 10;69(5-2):945–56. doi: 10.1016/j.neuron.2011.02.021 (PMC3057015; doi:10.1016/j.neuron.2011.02.021)
Supplement: Document S1. Three Figures [file mmc1.pdf]

Neuron, Volume *69*

Supplemental Information

A Critical Role for Neurofascin in Regulating Action Potential Initiation through Maintenance of the Axon Initial Segment

Barbara Zonta, Anne Desmazieres, Arianna Rinaldi, Steven Tait, Diane L. Sherman, Matthew F. Nolan, and Peter J. Brophy

Inventory of Supplementary Information:

Figures S1, S2 and S3

Movie S1 (see separate video)

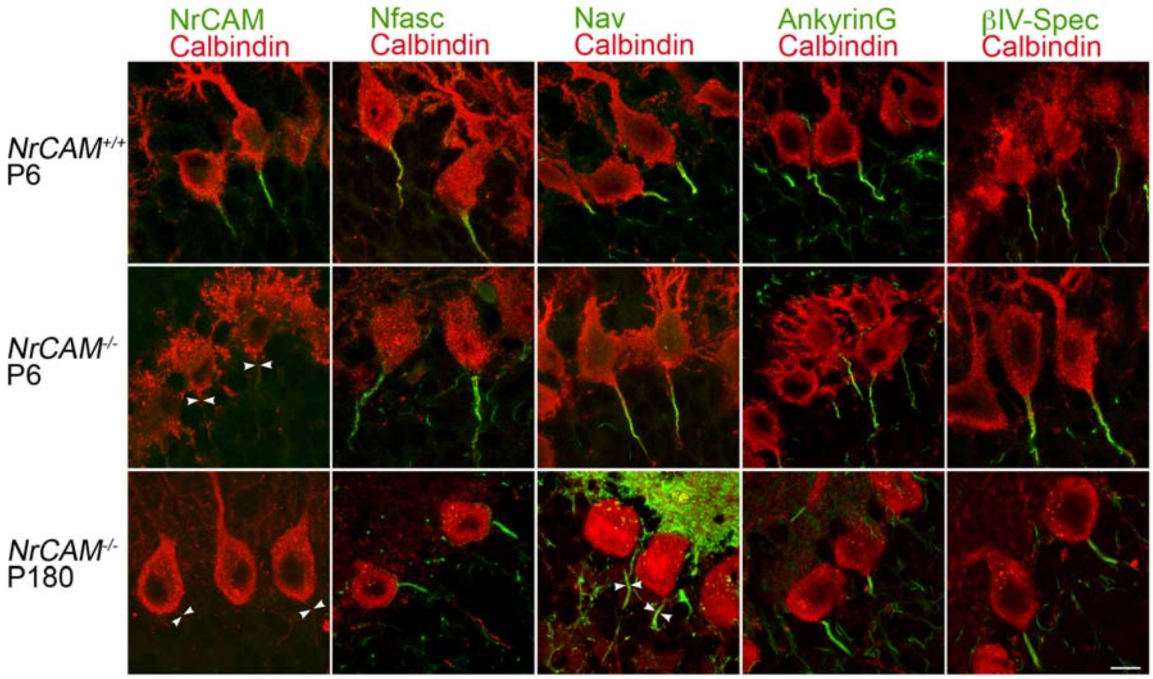

Figure S1, related to Figure 1. Ablation of *NrCAM* Affects neither the Assembly nor Stability of the AIS

Immunofluorescence of Calbindin-positive Purkinje cells from cerebella cryostat sections in NrCAM-null mice and wild-type littermates at post-natal day 6 (P6) showing that NrCAM is not required to target either Nfasc186 or other components of the AIS including Na_v channels and the cytoskeletal proteins AnkyrinG and βIV Spectrin. These proteins are still maintained at the AIS in NrCAM-null mice at P180 (arrowheads point to AIS). Scale bar, 10 μm

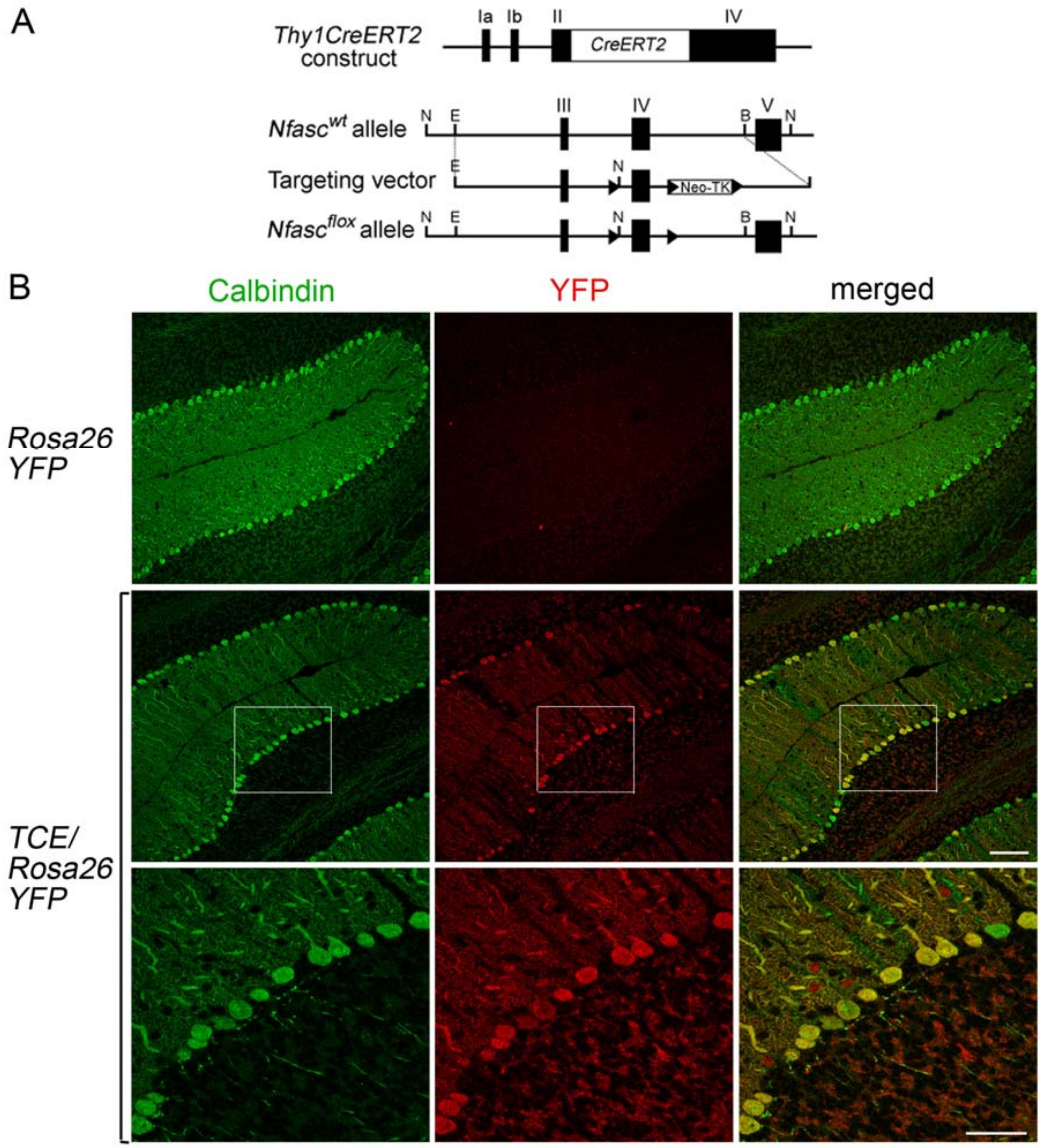

Figure S2, related to Figure 3. Characterisation of the *TCE* Transgenic Mouse

(A) Schematic diagram of the *Thy1CreERT2* (*TCE*) transgene, wild-type *Nfasc* gene, targeting vector and floxed allele after Cre-mediated excision of the PGK*neo*-HSV*tk* cassette. The CreERT2 cassette was inserted downstream of the Thy1.2 promoter for the expression of tamoxifen-inducible Cre specifically in neurons. The floxed allele of the

Nfasc gene contains two *loxP* sites flanking exon IV. Cre recombination of the floxed *Nfasc* allele leads to the deletion of exon IV resulting in a transcript with an in-frame stop codon in exon V leading to the known loss of Neurofascin protein expression (Sherman et al., 2005). N, NcoI; E, EheI; B, BamHI

(B) Immunofluorescence analysis of Cre activity in the cerebellum. Mice carrying the *TCE* transgene were mated with the *Rosa26YFP* reporter line to determine the expression and activity pattern of CreERT2 in the cerebellum. Analysis of YFP expression in *TCE/Rosa26YFP* mice 6 weeks after tamoxifen treatment shows strong expression of YFP in Purkinje cells (stained for Calbindin). Scale Bars : 100 μm (low power) and 50 μm (high power).

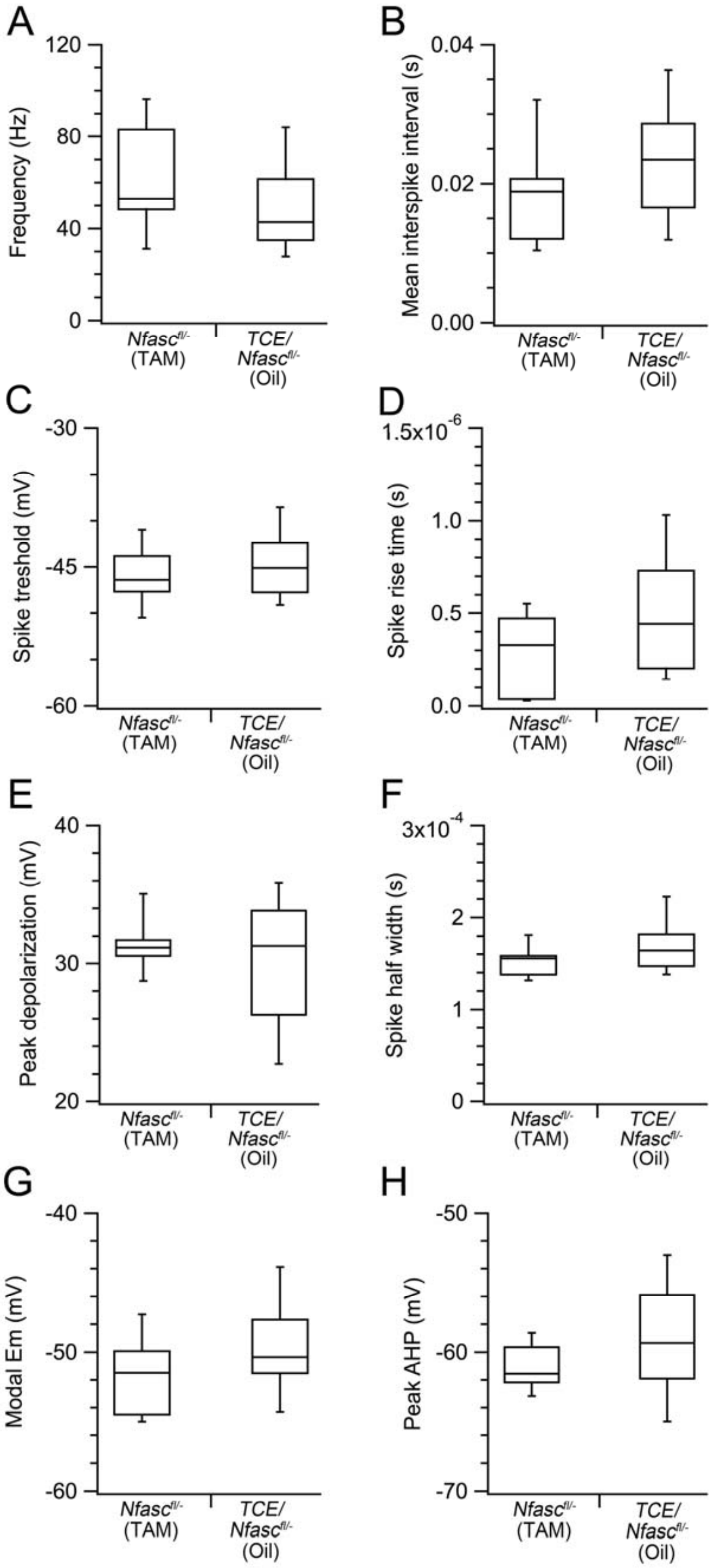
A
Frequency (Hz)
120
80
40
0
B
Mean interspike interval (s)
0.04
0.02
0.00
C
Spike treshold (mV)
-30
-45
-60
D
Spike rise time (s)
1.5x10^-6
1.0
0.5
0.0
E
Peak depolarization (mV)
40
30
20
F
Spike half width (s)
3x10^-4
2
1
0
G
Modal Em (mV)
-40
-50
-60
H
Peak AHP (mV)
-50
-60
-70
Nfasc^fl/- (TAM)
TCE/ Nfasc^fl/- (Oil)

Figure S3, related to Figure 6. Properties of Spontaneous Action Potentials in Purkinje Cells do Not Differ Between the Two Control Groups

(A-F) There was no difference between *Nfasc*$^{fl/-}$(TAM) (n=8) and *TCE/Nfasc*$^{fl/-}$(Oil) (n=10) mice in the mean frequency (A), the mean interspike interval (B), the threshold (C), the 10%-90% rise time (D), the peak depolarization (E) and the half-width of spontaneous action potentials (F) (t_{16}<1.6, P>0.1).

(G-H) There was also no difference in the modal membrane potential (G) and the peak of the afterhyperpolarization (H) (t_{16}<1.6, P>0.1).

In the box plots, the horizontal bar is the median, the boxes indicate the 25–75 percentile range, and the vertical lines indicate the 10–90 percentile range.
