# Supplementary figures and images for: A Critical Role for Neurofascin in Regulating Action Potential Initiation through Maintenance of the Axon Initial Segment
Source: Neuron. 2011 Mar 10;69(5-2):945–56. doi: 10.1016/j.neuron.2011.02.021 (PMC3057015; doi:10.1016/j.neuron.2011.02.021)

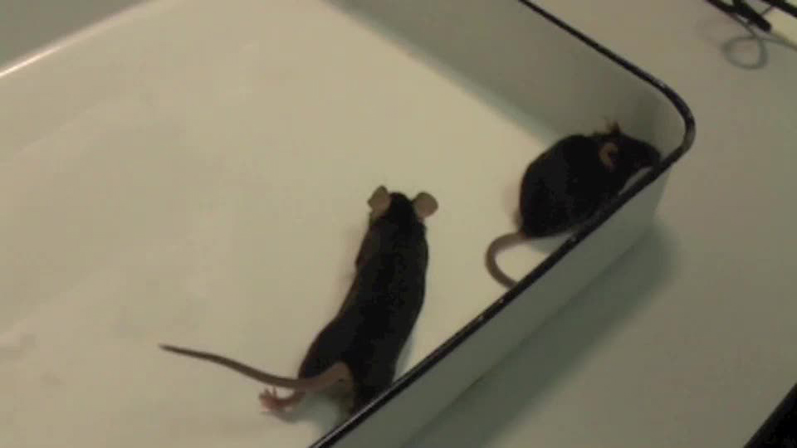

Supplement: Movie S1. Mice with a Disrupted AIS Have Altered Gait — At the beginning of the movie, the mouse on the left is a control littermate for the mouse on the right in which inactivation of the Neurofascin gene in neurons had been initiated 6 weeks earlier by tamoxifen administration. See also Figure 5. [file mmc2.jpg]
